# The G Protein-Coupled Glutamate Receptors as Novel Molecular Targets in Schizophrenia Treatment—A Narrative Review

**DOI:** 10.3390/jcm10071475

**Published:** 2021-04-02

**Authors:** Waldemar Kryszkowski, Tomasz Boczek

**Affiliations:** 1 General Psychiatric Ward, Babinski Memorial Hospital in Lodz, 91229 Lodz, Poland; waldemar.kryszkowski@gmail.com; 2 Department of Molecular Neurochemistry, Medical University of Lodz, 92215 Lodz, Poland

**Keywords:** schizophrenia, metabotropic glutamate receptors, positive allosteric modulators, negative allosteric modulators, drug development, animal models of schizophrenia, clinical trials

## Abstract

Schizophrenia is a severe neuropsychiatric disease with an unknown etiology. The research into the neurobiology of this disease led to several models aimed at explaining the link between perturbations in brain function and the manifestation of psychotic symptoms. The glutamatergic hypothesis postulates that disrupted glutamate neurotransmission may mediate cognitive and psychosocial impairments by affecting the connections between the cortex and the thalamus. In this regard, the greatest attention has been given to ionotropic NMDA receptor hypofunction. However, converging data indicates metabotropic glutamate receptors as crucial for cognitive and psychomotor function. The distribution of these receptors in the brain regions related to schizophrenia and their regulatory role in glutamate release make them promising molecular targets for novel antipsychotics. This article reviews the progress in the research on the role of metabotropic glutamate receptors in schizophrenia etiopathology.

## 1. Introduction

Schizophrenia is a common debilitating disease affecting about 0.3–1% of the human population worldwide [1]. Although no convincing evidence about its etiology has been presented so far, it is believed that it has a multifactorial origin involving the combinatorial influence of environmental, genetic, psychological, and social factors. The symptoms of schizophrenia are usually classified into three main groups: positive (e.g., auditory and visual hallucinations, disorganized thought, delusions, odd behaviors), negative (e.g., social withdrawal, flattened affect) and cognitive (e.g., dysfunctions in working memory, attention, visual and verbal learning). Cognitive deficits are among the core symptoms present in almost 98% of patients with schizophrenia [2]. They usually manifest prior to the onset of psychosis and persist, but evolve during disease development. The antipsychotics currently used in clinical practice can effectively treat positive symptoms, but they poorly address negative and cognitive alterations [3]. Moreover, an increasing number of patients discontinue medical treatment due to extrapyramidal side effects related to the use of first-generation typical antipsychotics or adverse metabolic effects induced by second-generation atypical drugs [4,5]. While most current antipsychotics act on dopaminergic and serotonergic systems, there is an urgent need to identify both new molecules and pharmacological targets with higher safety and efficiency toward core schizophrenia symptoms.

In recent decades, there has been an intensive investigation into the possible role of glutamate in the schizophrenia pathological processes. Glutamate is the main excitatory neurotransmitter in the brain, and its role is inextricably linked to the processes related to memory, cognition, or perception [6]. The prevailing glutamatergic theory of schizophrenia postulates hypofunction of N-methyl -D-aspartate receptor (NMDAR) as one of the factors contributing to pathophysiology of this disease [7]. The hypothesis of glutamate involvement is based on the observations of psychotomimetic effects of NMDAR antagonists—phencyclidine (PCP), MK-801 or ketamine, producing all three schizophrenia symptom clusters in healthy individuals [8,9]. These findings led to the clinical trials with several prospective drugs, for instance cycloserine or D-serine, positively modulating NMDAR activity [10,11], although their efficacy in schizophrenia was relatively modest.

In addition to NMDAR, AMPA and kainate receptors that mediate most of the excitatory neurotransmission in the central nervous system (CNS), glutamate is also a ligand for G protein-coupled metabotropic receptors. The important subtypes of theses receptors have been demonstrated to be relevant to the symptomology of schizophrenia and other psychiatric disorders such as anxiety and depression [12,13]. They have also been considered putative targets for a novel class of antipsychotics, and several preclinical and clinical trials have been aimed to test their utility and efficacy. Therefore, this review aims to provide an update on the current research status on glutamate metabotropic receptors in the treatment of schizophrenia.

## 2. Metabotropic Glutamate Receptors (mGluRs)—An Overview

**Figure 1:**
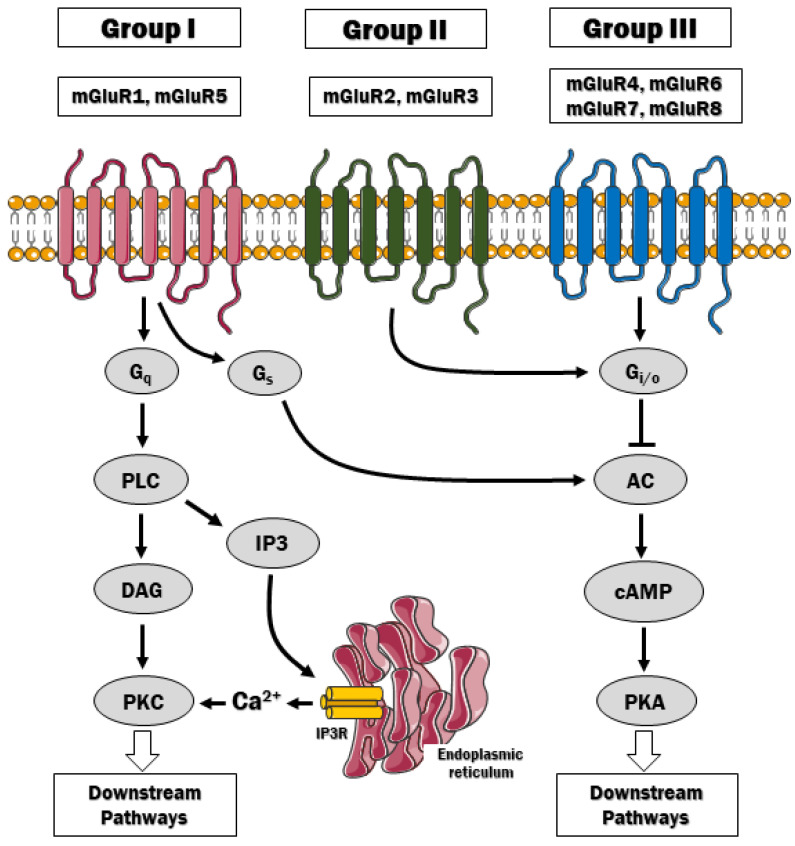
The signaling pathways downstream mGluRs. Group I mGluR is coupled to G_q_ protein, which stimulates phospholipase C (PLC) and the hydrolysis of phosphatidylinositol 4,5-bisphosphate (PIP_2_). PIP_2_ hydrolysis gives rise to inositol (1,4,5)-triphosphate (IP_3_) and diacylglycerol (DAG). The IP_3_ diffuses freely to the endoplasmic reticulum and activates the IP_3_ receptors to release Ca^2+^ to the cytosol. By contrast, mGluRs of group II and III classically couple to G_i/o_ proteins and inhibit adenylyl cyclase, thus affecting downstream signaling pathways via liberation of Gβγ.

Eight genes (GRM1 to GRM8) encoding mGluRs (mGluR1 to mGluR8) have been cloned and characterized, with multiple splice variants that are expressed in different cell types in the central and peripheral nervous system [14,15]. Up to date, the genetic loci for each receptor have been identified as well [15]. The mGluRs are divided into three groups I, II and III based on receptor structure, ligand selectivity and physiological action (Table 1). Group I includes mGluR1 and mGluR5, and their activation triggers phospholipase C-mediated effects. Group II, which includes mGluR1 and mGluR3, and group III involving mGluR4,6,7,8, are coupled to the inhibition of cAMP signaling via G_i_/G_o_ protein (Figure 1). In general terms, these receptors play a modulatory role by regulating neurotransmitter release, excitability, and synaptic plasticity. All glutamate receptors discussed here, except for mGluR6, which is primarily located in the retina, are expressed in neurons and glial cells. However, their distribution in cell subtypes may vary depending on the receptor.

## 3. Group I mGluRs

### 3.1. Biology of Group I mGluRs

In general, the mGluRs in group I (mGluR1 and mGluR5) are associated with the G_q_/G_11_-mediated signaling pathway and are strongly stimulated by L-quisqualic acid, which is thought to mimic the excitatory action of L-glutamic acid [19]. The classical pathway downstream these receptors includes activation of phospholipase Cβ, resulting in hydrolysis of plasma-membrane inserted phosphoinositide phospholipids and generation of inositol 1,4,5-trisphosphate (IP_3_) and diacylglycerol (DAG). IP_3_ diffuses to the ER, where upon binding to IP_3_ receptors, it allows for Ca^2+^ efflux and its mobilization in the cytosol. A growing body of evidence indicates that these receptors can also act through cascades downstream of G_q_, G_i/o_ and G_s_ or independent of G proteins [20,21,22,23]. These downstream effectors include, for instance, cyclin-dependent protein kinase 5 [24], extracellular signal-regulated kinase [25], c-Jun N-terminal kinase [26], casein kinase I [27], phospholipase D [28], mammalian target of rapamycin (mTOR)/p70 S6 kinase pathway or CREB (cAMP response element binding protein)-dependent signaling [29]. The coupling of these receptors to variety of signal-transducing pathways and their close association with NMDA receptors via scaffolding proteins Homer, SHANK, and GKAP-PSD95 [30] make them attractive targets for pharmacological interventions in schizophrenia.

Because mGluRs of group I are located predominantly postsynaptically, their activation results in neuronal depolarization, although mGluR1′s ability to elicit hyperpolarization was demonstrated in midbrain dopaminergic neurons [15]. Of relevance to schizophrenia, mGluR1/5 can influence both post- and presynaptic NMDA receptor currents, and this bidirectional regulation depends on calcium [31]. When intracellular Ca^2+^ concentration rises, activation of mGluR1/5 decreases NMDA receptor activity to provide control over abnormal, and potentially detrimental, Ca^2+^ increases. Group I mGluRs can also regulate postsynaptic excitability within the frontal cortex, thalamus, striatum, hippocampus and subthalamic nucleus, the brain structures associated with schizophrenia pathology [31,32]. Genetic and behavioral studies discussed below significantly contributed to our understanding of these receptors’ function in cognitive and sensomotoric processes relevant to schizophrenia.

### 3.2. mGluR1

The contribution of mGluR1 to schizophrenia was shown by postmortem studies which revealed increased expression of this receptor in the prefrontal cortex of schizophrenia subjects [33]. Further support was provided by Brody and Geyer, who demonstrated disruption of prepulse inhibition in mGluR1^-/-^ mice, which was ameliorated by mood stabilizer lamotrigine [34]. By contrast, mGluR1 antagonists selectively increased the basal level of prepulse inhibition in a mice model of sensorimotor gating impairment [35], a brain process that is typically altered in schizophrenic patients [36].

Genome-wide association studies identified 12 rare deleterious mutations in the GRM1 gene [37,38], and those mutations associated with schizophrenia also reduced mGluR1 signaling. However, the mutant receptors can be potentiated by series of small molecules developed by Cho and colleagues that have the efficacy to restore, at least partially, glutamate-mediated calcium signaling [39]. This suggests that schizophrenia deficits produced by these mutated receptors can be handled by mGluR1 positive allosteric modulators (PAM).

An increasing number of studies suggest that mGluR1 PAMs could be applied in treating positive and cognitive symptoms of schizophrenia what is consistent with the hypothesis of mGluR1 contribution to the regulation of long-term potentiation (LTP) [40,41,42]. Besides the potential utility of PAMs, it has been reported that also negative allosteric modulators (NAM) of mGluR1 can be efficient in reducing NMDAR-induced hyperlocomotion, deficits in prepulse inhibition, and social interactions in animal models of schizophrenia (Table 2) [42,43,44].

### 3.3. mGluR5

A growing body of evidence indicates that mGluR5 is an attractive pharmacological target to treat schizophrenia. CDPPB, the first mGluR5 PAM with favorable drug metabolism and pharmacokinetic properties, reversed amphetamine-induced hyperlocomotion and disruption of prepulse inhibition in rats (Table 2) [49]. Another mGluR5 agonist, VU0409551, produced rapid antipsychotic-like and cognition-enhancing activity in rodent models of schizophrenic symptoms. It selectively potentiated mGluR5 coupling to Gαq-mediated calcium mobilization and ERK1/2 signaling but not mGluR5 modulation of NMDA receptor currents in CA1 pyramidal cells [53]. This is in agreement with the ability of the drug to enhance long-term depression (LTD) at the Schaffer-Collateral-CA1 synapse induced by (*S*)-3,5-dihydroxyphenylglycine, a form of plasticity that requires ERK (extracellular signal-regulated kinase) activity [30].

VU0409551 is also useful in reversing the deficits in serine racemase (SR) knockout mice, in which the synthesis of D-serine is genetically ablated [55]. SR, the enzyme that produces the NMDA receptor co-agonist, has been recently identified as a risk gene for schizophrenia [11], and the SR^-/-^ mice mimic many behavioral and neurochemical abnormalities observed in this disease. VU0409551 was shown to enhance NMDA receptor function, rescue long-tern potentiation in hippocampal slices from SR^-/-^ mice and improve contextual fear memory [55]. Therefore, modulation of mGluR5 can be considered an effective mechanism to improve synaptic plasticity and memory impairments in schizophrenia.

## 4. Group II mGluRs

### 4.1. General Characteristics

Among mGluRs belonging to group II, the expression of mGluR2 is restricted to only a few brain regions including cerebellar cortex and the olfactory bulbs. The mGluR3 is more widely expressed and is extensively detected in dentate gyrus, cerebral cortex, striatum, cerebellar cortex, substantia nigra pars reticulata, olfactory tubercle and lateral septal nucleus [56]. In these structures, mGluR3 localization is either presynaptic, postsynaptic, or glial [57,58]. In contrast, mGluR2 is found only in neurons acting at the preterminal region away from the sites of neurotransmitter release [56]. Presynaptic mGluR2/3 are targeted to the membrane regions distant form the synaptic cleft, where they transduce signals induced by synaptic or astrocytic glutamate release [30].

In contrast to group I, glutamate receptors belonging to group II are mostly coupled to G_i/o_ proteins, which in turn inhibit cAMP signaling, certain calcium channels and other downstream pathways via G_βγ_ subunits. These receptors also couple extracellular glutamate signaling to MAPK and PI3 kinase activity, thus expanding the complexity of mechanisms by which they may contribute to synaptic defects in schizophrenia [15]. Moreover, as mGluRs 2/3 primary function to inhibit neurotransmitter secretion form various type of synapses (GABAergic, glutamatergic, dopaminergic, etc.), they have gained an attention as effective pharmacological targets for novel antipsychotics. It is now becoming apparent that these mGluRs do not act in isolation, rather they crosstalk with other mGluRs. For instance, formation of mGluR2/mGluR4 heterodimer is known to regulate the interaction of both receptors with allosteric ligands [59]. By contrast, mGluR3 supports mGluR5-dependent signaling in cortical pyramidal neurons without forming a heterodimeric complex [60].

### 4.2. mGluR2/3 Function in Schizophrenia

It has been demonstrated that activation of mGluR2/3 attenuates amphetamine-induced dopamine release in the dorsal striatum, nucleus accumbens and substantia nigra via a mechanism independent from vesicular release or de novo synthesis or reuptake of dopamine [61]. Pharmacological activation of these receptors also reduced some of the behavioral and cellular deficits of NMDA receptor hypofunction in animal models of schizophrenia. These included normalization of locomotor activity, reduction of the frequency of stereotypic behaviors [62,63] and improvement of working memory [62,64]. Presynaptic mGluR2/3 usually provide negative feedback on glutamate action. In line with that attenuation of glutamate release in the medial prefrontal cortex (mPFC) induced by NMDA receptor antagonist—ketamine was seen in the presence of mGluR2/3 agonists [65]. In glia, however, they potentiate synaptic glutamate uptake acting on glial glutamate transporters [66]. Postsynaptic mGluR2/3 receptors have also been localized to dendritic spines of hippocampal dentate granule cells in a close proximity to glutamatergic synapses [67] suggesting their role in increasing neuronal excitability, as was shown for mGluR3 in CA3 pyramidal neurons [67]. It is still unclear whether this enhancement (either NMDA receptor current or other currents) is associated with the antipsychotic potency of mGluR2/3 agonists but undoubtedly, they can provide normalization of glutamate levels and NMDA receptor function in schizophrenia.

A growing body of evidence also indicates that postsynaptic mGluR2/3 may play a pivotal role in the layer III circuits of the dorsolateral prefrontal cortex, the structure which is associated with executive functions including working memory and selective attention. The modulatory role of GluR2/3 toward glutamate release provides a rationale for developing selective PAMs and NAMs to counterbalance the excessive glutamate tone in the brain of schizophrenia individuals. However, as will be discussed in the subsequent sections, the outcome of clinical trials were mixed, highlighting the further need for understanding the contribution of GluR2/3 to disease development and progression.

Gonzalez-Maeso and colleagues reported that 5-HT2A serotonin receptor and mGluR2 are co-expressed in the same population of cortical neurons and are implicated in psychosis associated with schizophrenia [68]. This was based on the observation that 2AR-mGluR2 complexes targeted by hallucinogenic drugs activated unique cellular and behavioral response, which was blunted by the activation of mGluR2. In support, mGluR2 knockout mice were insensitive to behavioral effects of hallucinogenic drugs [69], further suggesting that 2AR-mGluR2 complex, but not 5-HT2A receptor alone, is obligatory for neuropsychological responses to hallucinogens. Postmortem analysis of untreated schizophrenic brains revealed up-regulation of 5-HT2A receptor and concomitant down-regulation of mGluR2, a pattern that could predispose to psychosis [68]. On the other hand, antagonism of 5-HT2A with atypical antipsychotics has been shown to affect mGluR2 expression in rodents [70], which may raise an issue about therapeutic efficacy of mGluR2 ligands in patients treated with atypical serotonergic antipsychotics.

### 4.3. Targeting mGluR2/3 in Schizophrenia Treatment

#### 4.3.1. Preclinical Studies

The potential use of mGluR2/3 agonists in the treatment of schizophrenia was heavily tested in pre-clinical studies using different animal models (Table 3).

The early report showed that administration of LY354740, a prototypical drug interacting with both mGluR2 and mGluR3, was effective in attenuation of deficits in working memory, locomotion and excessive glutamatergic signaling in PCP-induced model of NMDA receptor hypofunction [62]. In the same model, another mGluR2/3 agonist LY379268 reversed certain behavioral phenotypes and prepulse inhibition [74] to a degree comparable with atypical antipsychotic clozapine. Further studies on mGluR3 and mGluR2 knockout mice demonstrated that mGluR2, but not mGluR3, mediated the effects of LY379268 in experimental models predictive of antipsychotic activity [71]. LY379268 also showed promising effects in other schizophrenia models based on pharmacological blockage of NMDA receptor (ketamine and MK-801) [87] but had a marginal effect on amphetamine (AMPH)-induced hyperlocomotion in rats [72]. These results demonstrate the specificity of mGluR2/3 agonists toward glutamatergic signaling without any effects on the dopamine system.

Despite some conflicting results, recent studies supported the positive effects of mGluR2/3 stimulation on cognitive and negative symptoms of schizophrenia. LY379268 was found to be successful in reversing post-weaning social isolation-induced recognition memory deficits [88] and prenatal stress-induced schizophrenia-like neurochemical and behavioral changes. The latter is likely to be caused by increased expression of growth arrest and DNA damage 45-β (Gadd45-β) protein and subsequent epigenetic modifications. Increased binding of Gadd45-β to the promoter regions of reelin, BDNF, and GAD67 was also detected upon treatment with LY379268 [89], suggesting a putative underlying mechanism. Interestingly, Holloway and colleagues, using a model of variable and unpredictable stress in mice, showed decreased mGluR2-dependent antipsychotic-like effect of LY379268 in mice born to stressed mothers during pregnancy [90]. These data support the hypothesis of an early developmental origin of schizophrenia and indicate that modulation of mGluR2/3 may impact the epigenetic process in the early phase of this disease.

These studies’ critical question is whether early stage treatment can rescue cognitive defects and confer benefits for schizophrenia behavior in adulthood. Using neurodevelopment schizophrenia model, Xing and coworkers has recently reported that LY379268 can improve learning deficits in juvenile rats via a mechanism dependent on inhibition of glycogen synthase kinase-3β (GSK3β) [91]. Moreover, the drug affected neither excitability in the prefrontal cortex nor glutamatergic signaling but prevented dendritic spine loss in adults. Similarly, juvenile treatment with a subchronic dose of a novel mGLuR2 agonist/mGluR3 antagonist LY395756 alleviated the learning deficits and cognitive flexibility impairments in adults [92].

Studies on long-term administration and dose-dependent effects of mGluR2/3 agonists in normal and disease conditions are scarce. An early report suggested that LY379268 dose-dependent reduction in hyperlocomotion in PCP and AMPH-induced models of schizophrenia is not reproduced during chronic treatment [75]. In the drug abuse model, consecutive administration of LY379268 decreased toluene-induced hyperactivity [93] while it did not affect PCP-evoked hyperlocomotion when repeatedly dosed [94]. Subchronic treatment with LY354740 also failed to reverse ketamine-induced hyperlocomotion and prepulse inhibition deficits [95]. Comparison of short versus long-term (2 days vs. 14 days) LY379268 effects revealed a significant reduction in mGluR2 expression in the hippocampus, nucleus accumbens, caudate and ventral pallium as well as increased pERK/ERK ratio during 14-day drug administration [96]. The authors also observed increasing immunoreactivity of CREB protein across all brain regions, which may suggest the activation of multiply compensatory mechanisms in response to drug’s chronic systemic administration.

Subsequently, several other drugs have been developed. A prototypical mGluR2/3 PAM—LY487379 promoted cognitive flexibility, facilitated behavioral inhibition [97], and reversed social discrimination deficits induced by neonatal PCP treatment [78]. These effects were accompanied by enhanced extracellular serotonin and norepinephrine levels in the prefrontal cortex. Social recognition impairments elicited by MK-801 were also amended by LY379268 and mGluR2 PAMs—biphenyl indanone A (BINA) and TASP0443294 [41]. Since the effect of BINA on social memory was blocked by mGluR2/3 antagonist LY341495 [98], the positive effects of mGluR2/3 agonists seem to be mediated by mGluR2. BINA also modulated excitatory neurotransmission in mPFC as well as attenuated the effects of in vivo activation of 5-HT2A receptor and serotonin-induced increases in spontaneous excitatory postsynaptic currents in mPFC [99]. Like TASP0443294, other mGluR2 PAMs—JNJ-40411813 and ADX71149 inhibited PCP-induced hyperlocomotion but did not affect AMPH-elicited hyperactivity [100]. Recently, novel mGluR2 PAM—SAR218645 reduced head twitch behavior in several models of schizophrenia positive symptoms but was ineffective in prevention of hyperactivity in pharmacological and transgenic models [101]. However, in cognitive symptoms models, it improved MK-801-induced episodic memory deficits and reversed working memory impairments in NMDA receptor-deficient (Nr1^neo−/−^) mice, thus providing a piece of convincing evidence for mGluR2 PAMs cognition-enhancing effects in the genetic model of schizophrenia.

Given that antipsychotic drugs are administered chronically, it is unknown whether the acute effects of novel substances reported in animal studies will also be seen in clinical practice or their long-term use will result in tolerance. Furthermore, it must be considered that mGluR2/3 agonists, although successfully reversing some cognitive effects in NMDA receptor hypofunction models [63,87,88], have no effect or impair cognition in healthy animals [76]. This implies that the therapeutic use of mGluR2/3 agonists may be limited to the conditions of NMDA receptor dysfunction or have difference efficacy depends on the disease state.

#### 4.3.2. Clinical Trials

The first drug targeting mGluR2/3 receptors that has been clinically tested in human for the treatment of schizophrenia was LY-2140023, as it was suspected that it might normalize hyperactive cortical pyramidal neurons in the thalamus, prefrontal cortex and limbic system [102]. The first run of randomized phase II clinical trial demonstrated that 40 mg LY-2140023 taken twice daily improved both positive and negative, but not cognitive, symptoms of schizophrenia, as measured with PANSS (Positive and Negative Syndrome Scale) and CGI-S (Clinical Global Impression Scale) when compared to placebo. However, no significant difference was seen between tested group and olanzapine positive group. A second randomized, double-blind clinical trial showed no differences between various LY-2140023 doses (5, 20, 40, 80 mg) and placebo on PANSS total score [103]. In 2013, another phase II, randomized, parallel, active-controlled study was performed to investigate efficacy and long-term side effects of LY-2140023 administration [104]. Over the first 6–8 weeks of treatment, no significant difference was seen between LY-2140023 and olanzapine, risperidone, or aripiprazole groups, but at later time points, the effectiveness of antipsychotic medications was higher than LY-2140023. As no primary endpoint was met, the Eli Lilly company that developed the drug ceased their ongoing studies of phase III. The possible causes of failure were discussed by Li and colleagues in [105].

Addex Company announced that its newest mGluR2 PAM, ADX71149 demonstrated safety and tolerance in healthy men and women, and it passed IIa of clinical trial being efficient in patients with negative schizophrenia symptoms [106,107]. The drug at a dose of 150 mg administered twice daily for 7 days significantly ameliorated smoking withdrawal-evoked deficits in attention and episodic memory compared to placebo. At a higher dose (500 mg), ADX71149 reduced S(+) ketamine-induced negative symptoms and improved the total score of BPRS (Brief Psychiatric Rating Score) [107]. Despite these promising results, as of 2021, the results of phase III have not been disclosed to public.

In 2016, AstraZeneca released the phase II outcome on efficacy and safety of AZD8529, a selective PAM at the mGluR2 in symptomatic patients with schizophrenia [108]. There were no differences in PANSS total, negative, and positive symptoms subscale and CGI-S scores between patients in treated versus placebo groups. In early 2020, randomized, placebo-controlled, double-blind, and multisite study led by J. Lieberman re-investigated the clinical efficacy of pomaglumetad (POMA, LY-404,039) and TS-134 using blood oxygen level-dependent response [109]. Treatment with high-dose of POMA (320 mg/d for 10 days) significantly reduced ketamine-induced BPRS total symptoms but did not affect target engagement [110]. In contrast, TS-134 administered at a 20 mg/day dose for 6 days demonstrated both symptom reduction and target engagement.

Despite the progress that has been made in recent years, the mechanisms of mGluR2/3 action are still not elucidated, leaving the questions about effectiveness and safe window for mGluR2/3 agonist treatment unanswered. However, given the tolerance and low adverse effects, selective mGluR2/3 PAMs and agonists should still be considered promising drugs in schizophrenia treatment alone or in combination with other antipsychotics.

#### 4.3.3. Advantages and Disadvantages of Clinical Use of mGluR2/3 Agonists

It has been widely documented that typical antipsychotics may be associated with higher risk of extrapyramidal symptoms (EPS), whereas the use of atypical antipsychotics can results in higher risk of metabolic syndrome [111,112]. Clinical trials for mGluR2/3 agonists showed neglectable spectrum of side effects commonly seen for antipsychotics targeting dopaminergic system. LY2140023, for instance, was generally well tolerated, produced no changes in electroencephalogram and did not increase the treatment-emergent adverse events (TEAEs) [113]. Moreover, there were no withdrawal effects after 4-week drug administration [114]. The safety, tolerability, and potential side effects of ADX71149 in patients with schizophrenia were also a subject of clinical investigation. The aforementioned preliminary results of Phase IIa demonstrated a trend toward separation from placebo after 1 month of treatment when the drug was used as an adjunct to antipsychotics, thus suggesting a potential for ameliorating of negative symptoms [115].

Although no severe side effects were observed, the efficiency of mGluR2/3 agonists used as a monotherapy in schizophrenia turned out to be comparable to currently used antipsychotics. In a phase II comparative safety study, LY2140023 did not bring any statistically significant improvement over olanzapine, risperidone or aripiprazole in terms of positive or negative symptoms [104]. The initial improvement in PANSS total score as well as the incidence of serious adverse effects were also comparable between groups. In 24-week randomized, double-blind study, LY2140023 demonstrated lower improvement in PANSS total score and higher incidence of serious adverse effects compared to aripiprazole and lack of significant changes in the incidence of TEAEs and suicidal behavior [116]. Despite these disappointing results of mGluR2/3 agonists as a monotherapy, the drugs may still have potential as an adjunct therapy to currently used antipsychotics [115].

## 5. Group III mGluRs

### 5.1. General Characteristics

Group III of metabotropic glutamate receptors is the most extensive family consisting of mGluR4, mGluR6, mGluR7 and mGluR8 subtypes. Similar to group II, these receptors signal via Gα_i/o_ to inhibit adenylyl cyclase [15] and module the activity of several downstream signaling pathways, including MAPK and PI3-kinase [17]. Activation of mGluRs of group III can regulate neurotransmitter secretion by acting on several ion channels or through G_βγ_-dependent inhibition of vesicular fusion [117]. Their contribution to the induction of two forms of short-term synaptic plasticities: paired-pulse facilitation and post-tetanic potentiation, has also been demonstrated [118,119]. The function of mGluR6 will not be discussed here due to its restricted expression in retinal ON bipolar cells, in which it amplifies visual transmission [120].

### 5.2. mGluR4

mGluR4 is expressed in the cerebellar cortex, basal ganglia, thalamus, and hippocampus, predominantly in the presynaptic active zones. In the cerebellar cortex and hippocampus, mGluR4 localizes to the terminals forming type I synapses, while in basal ganglia its location is mostly in type II synapses in dendritic shafts. In the hippocampus, mGluR4 labeling was predominant in the dentate molecular layer and CA1-3 stratum lacunose molecular [121]. Still little is known about the presynaptic mechanisms of mGluR4. The most compelling physiological function evidence has been demonstrated at the glutamatergic synapses between parallel fibers and Purkinje cells. Activation of mGLuR4 at these synapses was exclusively responsible for the depression of parallel fiber-Purkinje cells excitatory transmission and inhibition of presynaptic Ca^2+^ entry [122,123]. The mGluR4 receptors are also expressed in GABAergic and glutamatergic synapses in brain regions involved in acoustic startle function. As startle reactivity is modulated by cortico-striato- pallido-pontine circuity [124], it is hypothesized that glutamate release from the auditory afferents may be inhibited by the presynaptically located group III mGluRs. Alterations in the presynaptic release of GABA and/or glutamate, especially in the hippocampus and basal ganglia, may account for decreased acoustic startle response and prepulse inhibition in mGluR4^-/-^ mice [125]. A much more significant increase in glutamate levels was observed in the caudate nucleus, which receives extensive glutamatergic innervation from the cerebral cortex, of mGluR4^-/-^ mice stimulated with NMDA [126]. Since the activation of mGluR4 reduces glutamatergic transmission in the hippocampus [127], prepulse inhibition and acoustic startle response deficits resulted from mGluR4 knockout may be associated with NMDA receptor activity. Disrupted prepulse inhibition and acoustic startle response have been found in various neuropsychiatric diseases, including schizophrenia [128,129].

#### Targeting mGluR4 in Preclinical Models of Schizophrenia

Investigation the utility of individual representatives of group III mGluRs in schizophrenia treatment became possible since the discoveries of blood brain barrier permeable selective ligands suitable for in vivo studies (Table 4). This enabled the first reports on the antipsychotic activity of LSP1-2111 and LSP4-2022, which behaved as preferential orthosteric agonists of mGluR4 [130]. These compounds showed improved selectivity, as their affinity for mGluR4 was 30- and 300-fold higher than for mGluR8, respectively. LSP1-2111 reversed amphetamine- and MK-801-induced hyperlocomotion as well as DOI (2,5-dimethoxy-4-iodoamphetamine)-induced head twitches [131]. The efficacy of LSP1-2111 was also demonstrated in various preclinical models of cognitive and negative schizophrenia symptoms including object recognition and social interaction tests [132]. Similarly, LSP4-2022 has been shown to attenuate MK-801-evoked neurotransmitter release and possess antipsychotic-like activity, which was abolished by pharmacological blockers of 5-HT1A receptors [133]. Further studies revealed that LSP4-2022 administered at subthreshold dose produced antipsychotic effects acting synergistically with 5-HT1A receptor agonist—8-hydroxy-dipropylaminotetraline and agonists of GABAB receptors [134]. A robust antipsychotic effect was also seen when LSP4-2022 was administered together with M4 muscarinic receptor PAM—VU152100. One of the results of this combination was a reduction of 5-HT2A-dependent spontaneous excitatory postsynaptic currents in the prefrontal cortex, suggesting cooperative action of mGluR4 and M4 receptors in glutamate release [135].

Similar promise in alleviating of all three symptom clusters of schizophrenia was provided by mGluR4-selective PAMs—LuAF21934, LuAF32615 and ADX88178. In behavioral tests modeling positive, negative, and cognitive symptoms, these compounds displayed antipsychotic action, albeit with different efficiency. LuAF21934 and LuAF32615 reversed MK-801- and amphetamine- induced hyperactivity in a dose-dependent manner [136]. Furthermore, both compounds inhibited DOI-induced head twitches and normalized increased frequency of spontaneous excitatory of postsynaptic currents in brain slices. However, Lu AF21934 was unable to antagonize DOI-induced behavioral deficits in mGluR4^-/-^ mice. Both tested drugs reversed deficits in social interactions and were active in the delay spatial alternation test, thus providing a support for a potential utility of pharmacological targeting of mGluR4. ADX88178 was effective in reduction of MK-801-induced hyperlocomotion and DOI-evoked head twitches but did not show any effects in conditioned avoidance response test in rats [137].

Recently, Fazio and colleagues showed that cinnabarinic acid, a metabolite of kynurenine pathway of tryptophan metabolism, acts at low doses as a partial agonist of mGluR4, with no activity at other mGlu receptor subtypes [138]. Systemic treatment with a dose < 1 mg/kg attenuated MK-801-evoked glutamate release and hyperlocomotion [139]. Cinnabarinic acid also demonstrated a dose-dependent effect in several behavioral tests used to score antipsychotic activity, but inhibited excitatory synaptic transmission and no longer exerted antipsychotic activity in mGluR4^-/-^ mice. Interestingly, the level of cinnabarinic acid was significantly reduced in the prefrontal cortex of schizophrenia individuals [139].

### 5.3. mGluR7

The mGluR7 is highly expressed in brain regions responsible for emotion, cognition, and reward, hence in the hippocampus, amygdala, dorsal striatum, nucleus accumbens, amygdala, locus coeruleus, olfactory bulbs and ventral tegmental area [142]. Presynaptically localized mGluR7s acting as autoreceptors are mainly involved in regulating glutamate secretion, while those functioning as heteroreceptors regulate GABA release and possibly other monoamines [143,144]. Because these receptors are immobilized at presynaptic active zones of both excitatory and inhibitory synapses, they play a critical role in shaping synaptic response for GABAergic and glutamatergic neurotransmission [145,146]. Compared to other group III receptors, mGluR7 has a low affinity for glutamate; therefore it can be activated only by sufficiently high levels of extracellular glutamate [147]. For that reason, it is assumed that mGluR7 is activated only during intense, high-frequency synaptic stimulation. On the other hand, the newest results suggest that mGluR7 may be constitutively active and that activity can be sustained by the interaction with postsynapic adhesion molecule Elfn2 at the excitatory synapses [148,149]. Mice lacking mGluR7 demonstrated deficits in short-term neural plasticity in the hippocampus and some impairments in memory and learning [150]. Recent studies also showed that mGluR7^-/-^ mice displayed extinction of a conditioned fear response [151] and reduced shock-induced freezing [152], which are amygdala-dependent paradigms. Moreover, mice exhibited reduced anxiety-like responses consistent with mGluR7 role in emotional disorders [153]. Recently, Tassin and coworkers showed that inactivation of this receptor modulated the global thalamic excitability, decreasing its propensity to switch from tonic to oscillatory mode [154]. Therefore, mGluR7-dependent reduction in thalamocortical neurotransmission, a circuit suggested to be overactive in schizophrenia, may be a goal for new therapies of this disease.

#### Targeting mGluR7 in Preclinical Models of Schizophrenia

The first selective mGluR7 agonist—AMN082 was discovered in 2005 using a random high-throughput screening of chemical libraries [155] (Table 4). A few years later, Wieronska and colleagues demonstrated that it had no effect on amphetamine-induced hyperlocomotion, but rather it produced an enhancement in MK-801-induced hyperactivity [131]. Using a behavioral model of hallucinations, the same authors showed increased DOI-induced head twitches. In this experiment, hallucinogenic-like activity was achieved by administering the 5-HT2A receptor agonist DOI, which is known to provoke a characteristic behavior of twitches of heads in mice [156]. AMN082-potentiated hyperlocomotion and head twitches were absent in mGluR7^-/-^ mice [131], suggesting that mGluR7 mediated them. These results provided evidence for lack of antipsychotic activity and instead showed the pro-psychotic action of AMN082 in preclinical models. However, several studies suggested more complex activity of this drug with the observable effects detected in some systems but not in others [146]. Therefore, the negative findings with AMN082 in the pharmacological animal models of schizophrenia should be interpreted with caution.

So far, only two mGluR7 NAMs—MMPIP and ADX71743 have been tested in schizophrenia models [157,158]. Cieslik and coworkers used MK-801-induced hyperactivity and DOI-induced head twitches to test both drugs’ efficacy in the amelioration of positive symptoms of schizophrenia [140]. MMPIP and ADX71743 reversed MK-801-induced deficits; however, ADX71743 was effective at lower doses (5 mg/kg), while MMPIP was active only at the highest administered dose (15 mg/kg). This contrasts with the previous study showing only a small reduction in amphetamine-induced hyperactivity by ADX71743, and much higher doses (100 and 150 mg/kg) were necessary to observe a statistically significant effect [158]. Both drugs also reversed DOI-induced head twitches. Again, the activity of ADX71743 was observed at doses lower than that for MMPIP (2.5 mg/kg and higher vs. 5 mg/kg and higher). In the study of Kalinichev, ADX71743 was active at the dose of 100 mg/kg and higher [158]. Both drugs were also active when tested in models of cognition, attentional deficits and negative symptoms of schizophrenia [140]. Several other drugs targeting mGluR7 have been synthesized recently, for instance VU6010608 (2017) or VU6027459 (2020), but their utility in schizophrenia treatment has not been investigated yet.

### 5.4. mGluR8

The mGluR8 was cloned as the last receptor in the mGluR family [159]. It is widely expressed throughout the CNS, especially in the olfactory bulb, lateral reticular nucleus of the thalamus, cerebral cortex, cerebellum, hippocampus and retina [160]. Immunochemical studies demonstrated that mGluR8 is located in the terminal fields of the lateral perforant path in the outer molecular layer of the dentate gyrus and the CA3 stratum lacunosum. However, its highest level was observed in the caudate nucleus and putamen, the critical brain regions linked with schizophrenia. Electrophysiological studies using selective agonists suggest that mGluR8 may function as a presynaptically localized autoreceptor gating synaptic transmission into the hippocampus [161]. Therefore, the receptor may be activated only by high concentrations of released glutamate. In such circumstances, mGluR8 may provide negative feedback for glutamate synthesis, packaging and/or release from synaptic boutons [161,162].

By analyzing mGluR8 null mutant mice’s performance, Gerlai and coworkers demonstrated subtle behavioral alterations involving novelty-induced hyperactivity and delayed response to certain stimuli [163]. By contrast, Goddyn and colleagues did not find any deficits in neuromotor performance, prepulse inhibition, social exploration, learning and memory in mGluR8^-/-^ mice compared to wild type [125]. Although the behavioral effects of mGluR8 knockout may be inconclusive, there is an agreement on its function in controlling body weight [126,164,165]. The mGluR8^-/-^ mice have also been shown to demonstrate anxiety phenotype [160,166]. However, some newest studies failed to reproduce this finding [164,165]. Despite these discrepancies, the role of mGluR8 in memory and learning suggests that modulation of its activity by specific agonists/antagonists may be beneficial for the treatment of cognitive alterations in schizophrenia.

#### Targeting mGluR8 in Preclinical Models of Schizophrenia

Several mGluR8 ligands have been identified, and their antipsychotic properties have been tested in various animal models (Table 4). (R,S)-4-Phosphophenylglycine (PPG), a selective agonist of group III mGluRs with 10-fold higher affinity for mGluR8 than for mGLuR4, 6, 7 [167] depressed field excitatory postsynapic potentials (fEPSP) of the lateral entorhinal cortex to hippocampal granule cell projection [168] and significantly reduced neurotransmitter release without affecting presynaptic Ca^2+^ entry. Interestingly, PGG was also able to act as an inverse agonist at mGluR7 [169]. Another phenylglycine derivative, (S)-3,4-dicarboxyphenylglycine (DCPG), with the selectivity for mGLuR8 100-fold higher than for other groups’ III members, decreased amphetamine but not PCP-induced hyperactivity, but at this dose (2.5 mg/kg) did not affect spontaneous locomotor activity [141]. This contrasts with other studies showing significant reversal of amphetamine-induced locomotor activity; however, the discrepancy may arise from dose-dependent inhibition of spontaneous locomotor activity by the drug, thus producing confounding interpretation [141,160]. As no alterations in prepulse inhibition were seen in mGluR8^-/-^ mice, it may be possible that mGluR8 is not directly engaged in schizophrenia neuropathology. On the other hand, its role in hippocampal neurotransmission suggests that it may still be a promising target for novel antipsychotic with cognitive-enhancing properties. This is undoubtedly tempting but also requires experimental verification.

## 6. Concluding Remarks

A strong line of evidence from the preclinical studies indicates that representatives of all three groups of mGluRs should be considered when designing novel therapeutical strategies in schizophrenia treatment. The distribution of mGluR subtypes in brain regions traditionally linked with schizophrenia deficits, their modulatory action on other disease-associated receptors such as NMDA, AMPA or GABA(A) as well as the efficacy of their agonists or PAMs provide a strong foundation for considering mGluRs as potential targets for new generation antipsychotics. The research on animal models of schizophrenia demonstrated a crosstalk between various mGluRs ligands and dopaminergic, serotonergic, and glutamatergic pathways, thus revealing their potential to become effective alternative or improvement for the drugs currently used in clinical practice. This review has some limitations that should be acknowledged. First, it mostly covers preclinical findings due to limited availability regarding the efficiency of potential drugs in human studies. Second, whenever possible, we attempted to present the results of clinical trials, which sometimes led to confounding results due to lack of sample representativeness and inappropriate cohort selection. Third, the exact mechanism of potential mGluR-based novel antipsychotics is unknown, so the discussion about the molecular changes downstream particular mGluRs is frequently speculative. Lastly, the neuropharmacology of schizophrenia is rapidly changing, so although we tried to present the progress in the field by covering the majority of recently published papers, some of them could be omitted due to space restrictions. Even despite these limitations, the existing literature strongly supports further investigations into targeting mGluRs in schizophrenia. However, extensive research into how to translate preclinical results into clinically effective strategies is needed.

## Figures and Tables

**Table 1:** General properties of mGluR family. Genes encoding eight mGluR subtypes have been identified, many with multiple splice variants described here as a-f. mGluRs are subclassified into three groups based on sequence homology, synaptic localization and mechanism of action. Group I includes mGluRs 1 and 5, Group II includes mGluRs 2 and 3, and Group III includes mGluRs 4, 6, 7, and 8.

Group	Receptors/Splice Variants	Gene	Synapse Site	Expression in the CNS	Mechanism of Action
Group I	mGluR1 a,b,c,d,e,f	GRM1	Mainly postsynaptic	Abundant in neurons and taste buds	PLC-dependent Ca^2+^ mobilization, stimulation of adenylyl cyclase (certain systems), activation of MAP kinases, PLD, mTOR/p70 S6 kinase
	mGluR5 a,b	GRM5		Abundant in neurons and astrocytes	
Group II	mGluR2	GRM2	Presynaptic and postsynaptic	Abundant in neurons	Inhibition of adenylyl cyclase and voltage-dependent Ca^2+^ channels, activation of voltage-dependent K^+^ channels [16], MAPK and PI3 kinase pathway [17]
	mGluR3 GRM3A2 GRM3A4 GRM3A2A3	GRM3		Abundant in neurons and astrocytes	
Group III	mGluR4	GRM4	Mainly presynaptic (mGluR6 is postsynaptic in ON bipolar retinal cells)	Abundant in neurons (high in cerebellum) and taste buds	Inhibition of adenylyl cyclase, inhibition of voltage-dependent Ca^2+^ channels, activation of K^+^ channels [16], activation of cGMP phosphodiesterase [18] (mGluR6), MAPK and PI3 kinase pathway [17]
	mGluR6 a,b,c	GRM6		Retina	
	mGluR7 a,b,c,d,e	GRM7		Abundant in neurons	
	mGluR8 a,b,c	GRM8		Abundant in neurons (expression lower than mGluR4/7)	

**Table 2:** Summary of the preclinical studies on mGluR1/5 ligands.

Target	Tested Drug	Animal Model	Results	References
mGluR1	CFMTI	ketamine- and metAMPH-induced model	CFMTI reduced ketamine- and metAMPH-induced hyperlocomotion and drug-induced deficits in prepulse inhibition	[45]
	CFMTI	MK-801-induced model	Ameliorated MK-801-disrupted social interactions	[45]
	FTIDC	metAMPH-induced model	Reduced metAMPH-evoked hyperlocomotion and ameliorated deficits in prepulse inhibition	[43]
mGluR5	MPEP	PCP-induced model	Potentiated the psychotomimetic, cognition impairing and prepulse inhibition-disruptive effects of PCP.	[44,46]
	CHPG	MK-801-induced model Ketamine-induced model	CHPG reversed the cognitive-impairing effects of NMDA receptor antagonists and attenuated ketamine-induced locomotor activity, motor coordination and deficits in prepulse inhibition	[47,48]
	CDPPB	AMPH-induced model	Reduced AMPH-induced hyperlocomotion and ameliorated AMPH-disrupted prepulse inhibition	[49]
	CDPPB	MK-801-induced model	Attenuated MK-801-induced decrease in sucrose preference and deficits in cognitive flexibility	[50,51]
	ADX47273	PCP- and AMPH-induced model	ADX47273 blocked PCP- and AMPH-induced locomotor activity and decreased extracellular dopamine level in the nucleus accumbens	[52]
	VU0409551	AMPH-induced model MK-801-induced model	Reduced AMPH- and MK-801-induced hyperlocomotion	[53,54]

**Table 3:** Summary of the preclinical studies on mGluR2/3 ligands.

Tested Drug	Animal Model	Results	References
LY379268	PCP- and AMPH-induced model	LY379268 reversed PCP and AMPH-induced hyperactivity and PCP-evoked behavioral deficits	[71]
LY354740 LY379268	PCP- and AMPH-induced model	LY354740 and LY379268 attenuated PCP- but not AMPH-induced motor behaviors	[72]
LY379268	AMPH-induced model	LY379268 attenuated AMPH-induced ambulations and rearing but not fine motor movements	[73]
LY379268	PCP-induced model	LY379268 reduced PCP-induced falling, turning, and back pedaling in a dose-dependent manner but did not affect PCP-evoked forepaw treading	[74]
LY487379 LY379268	PCP- and AMPH-induced model	LY487379 and LY379268 induced dose-dependent reductions in PCP- and AMPH-induced hyperlocomotor activity. LY487379 reversed AMPH-induced disruption of prepulse inhibition of the acoustic startle reflex. LY379268 when administered chronically failed to block AMPH- and PCP-induced hyperlocomotor activity.	[75]
LY354740	PCP-induced psychosis	LY354740 induced anxiolytic-like effects and attenuated PCP-induced hyperlocomotion. It did not modify PCP-induced working memory deficits and had no effect on PCP-evoked amnesia.	[76]
LY354740	PCP- and AMPH-induced model	LY354740 moderated effects of PCP on prepulse inhibition of acoustic startle	[77]
LY354740 LY487379	PCP-induced psychosis	Acute pretreatment with LY354740 or LY487379 facilitated social discrimination in rats with PCP administration history without affecting total time spent in social interaction	[78]
LY379268	PCP- and MK-801-induced model	LY379268 blocked PCP- and MK-801-induced hyperlocomotion in dopamine-deficient and control mice	[79]
LY354750 LY544344	PCP-induced model	LY544344 but not LY354740 inhibited PCP-induced hyperlocomotion.	[80]
LY379268	Ketamine-induced model	LY379268 reduced ketamine-evoked hyperlocomotion but it failed to restore prepulse inhibition deficits. Low dose (1 mg/kg) produced anxiolytic effects whereas a higher dose (3 mg/kg) appeared to be anxiogenic.	[81]
LY379268	MK-801-induced model	LY379268 recovered the disrupted NMDA receptor expression induced by MK-801, enhanced NMDA-induced current in prefrontal neurons and reversed MK-801-induced NMDA receptor dysfunction via Akt/GSK-3β signaling.	[82]
LY379268	Ketamine-induced model	Pretreatment with either systemic or local LY379268 blocked ketamine-induced glutamate, but no dopamine release in the mPFC. Systemic but not local administration blocked the effect of NMDA on evoking glutamate release.	[65]
LY379268 LY341495	PCP- and LSD-induced model	LY341495 potentiated LSD-induced stimulus control, which was diminished in the presence of LY379268. In PCP-trained rats, LY341495 has no effect on stimulus control by an intermediate dose of PCP. In contrast, the training dose of PCP was significantly but incompletely antagonized by LY379268.	[83]
LY379268	Ketamine-induced model	LY379268 reversed ketamine-induced hyperactivity and inhibited ketamine-evoked norepinephrine release in the ventral hippocampus.	[84]
LY379268	PCP-induced model	LY379268 blocked PCP-evoked ambulatory activity and fine movements.	[85]
LY379268	two-hit model (PCP-induced on the background of neuregulin 1 mutation)	PCP significantly reduced NMDA receptor and GABAA receptor binding density in the prefrontal cortex, hippocampus, and nucleus accumbens while LY379268 restored NMDA and GABAA receptors level	[86]
LY379268	MK-801-induced model	LY379268 failed to improve MK-801-induced impairments in working memory. LY379268 augmented MK-801 potentiated gamma and high gamma oscillations but did not affect auditory-evoked gamma oscillation deficits caused by MK-801.	[87]

**Table 4:** Summary of the preclinical studies on Group III receptor ligands.

Target	Tested Drug	Animal Model	Results	References
mGluR4	LSP1-2111 LSP4-2022	MK-801-induced model AMPH-induced model	Reversed AMPH- and MK-801-induced hyperlocomotion	[131,134]
	LSP1-2111 LSP4-2022	DOI-induced model of hallucinations	Antagonized DOI-induced head twitches	[131,134]
	LuAF21934 LuAF32615	MK-801-induced model AMPH-induced model	Both compounds antagonized AMPH- and MK-801-induced hyperlocomotion	[136]
	LuAF21934 LuAF32615	DOI-induced model of hallucinations	Inhibited DOI-induced head twitches. Lu AF21934 was ineffective in mGluR4^-/-^ mice	[136]
	ADX88178	MK-801-induced model DOI-induced model of hallucinations	ADX88178 reduced MK-801-induced hyperlocomotion and DOI-evoked head twitches but had no effect in conditioned avoidance response test	[137]
	Cinnabarinic acid	MK-801-induced model	Reduced MK-801-induced glutamate release and hyperlocomotion	[139]
mGluR7	AMN082	MK-801-induced model AMPH-induced model	AMN082 did not influence AMPH-induced hyperlocomotion but enhanced MK-801-induced hyperactivity	[131]
	AMN082	DOI-induced model of hallucinations	Increased number of the DOI-induced head twitches	[131]
	MMPIP ADX71743	MK-801-induced model	Both drugs inhibited MK-801-induced hyperactivity, reversed MK-801-induced disturbances in novel object recognition, prepulse inhibition and spatial delayed alternation	[140]
	MMPIP ADX71743	DOI-induced model of hallucinations	Inhibited DOI-induced head twitches	[140]
mGluR8	DCPG	PCP-induced model AMPH-induced model	Decreased amphetamine but not PCP-induced hyperactivity	[141]
